# Neonatal sepsis is associated with behavioral abnormalities in very low birthweight infants at preschool age

**DOI:** 10.3389/fped.2022.906379

**Published:** 2022-07-18

**Authors:** Vito Giordano, Sophie Stummer, Claudia Lindtner, Renate Fuiko, Angelika Berger, Karin Pichler

**Affiliations:** Division of Neonatology, Pediatric Intensive Care and Neuropediatrics, Department of Pediatrics, Comprehensive Center for Paediatrics (CCP), Medical University of Vienna, Vienna, Austria

**Keywords:** sepsis, behavior, development, CBCL, NICU, preterm infant

## Abstract

**Objective:**

This study aimed to investigate neonatal sepsis as potential risk factor for adverse behavioral outcome in very low birth weight infants (VLBWI) at preschool age. Regardless of improvements in the obstetric and neonatal intensive care, preterm infants are still at high risk for behavioral problems later in life. The spectrum, origin and potential risk factors of these behavioral problems have not been well-defined.

**Methods:**

In this retrospective observational study, the influence of culture-proven neonatal sepsis on the behavioral outcome of VLBWI born at a gestational age <32 weeks was analyzed at 5 years of age in a multivariable regression model. Behavior was assessed with the Child Behavior Checklist (CBCL). Neonatal morbidities, socioeconomic status and neurodevelopmental outcome served as covariates in the analysis.

**Results:**

312 VLBWI entered the final analysis, of whom 11% had experienced neonatal sepsis. Neonatal sepsis appeared to be a relevant risk factor for both internalizing, i.e., emotional reactivity and anxiety/depression, as well as externalizing behavioral problems, i.e., oppositional and aggressive behavior in this cohort of VLBWI. Low socioeconomic status and male gender were additional statistically significant risk factors for both internalizing and externalizing behavioral problems. No difference in neurocognitive development was observed between the groups.

**Conclusion:**

The study supports the fact that VLBWI are vulnerable to multiple behavioral disorders independent of their cognitive development. In contrast to former assumptions, the results of the study emphasize that not only post-natal environment but also neonatal morbidities, especially neonatal sepsis, have an impact on behavioral outcome of VLBWI at preschool age.

## Introduction

Regardless of the improvements in the obstetric and neonatal intensive care within the last decade, with substantial increase in the survival of extremely preterm neonates, these infants are still at a high risk of neurodevelopmental impairment (NDI) (1).

In fact, ~30–40 percent of very low birth weight infants (VLBWI, birth weight <1,500g) have some degree of neurodevelopmental impairment (2–4) and need special healthcare resources (e.g., physiotherapy, speech therapy, occupational therapy) at 5 years of age (4). NDI is a composite and typically includes cognitive, motor and sensory impairments.

An emerging body of evidence suggests that not only NDI but also behavioral problems are a major issue in these individuals. The origin of behavioral problems in preterm infants is not fully understood yet. So far, most studies hypothesized that behavioral problems in preterm infants are associated with NDI and environmental factors, such as parental level of education and socioeconomic status (5–8). Indeed, environmental factors have been proven to be crucial factors influencing the behavioral outcome of preterm infants (6–10). However, behavioral problems do not seem to necessarily be associated with NDI in preterm infants (5, 6). Neonatal morbidities as potential risk factors for later behavioral problems in preterm infants have rarely been studied. Bartal et al. (7) investigated major brain injury and low birth weight as potential risk factors for adverse behavioral outcome in very preterm infants, but failed to find any significant relationship. Similarly, the EPIPAGE study did not find any correlation between intraventricular hemorrhage and behavioral problems in very preterm infants (5). In addition to brain injury due to intraventricular bleeding or hypoxic-ischemic events, it is now widely accepted that also perinatal infectious and inflammatory episodes are key risk factors for NDI (11). Inflammation may interfere with the regulation of neuronal migration and differentiation as well as synaptogenesis, thereby altering the fragile process of central nervous system development in preterm infants (11). Furthermore, it was shown that inflammation due to sepsis may directly affect myelinization and in turn white matter tissue, brain growth and attentional performance in preterm infants (12). VLBWI are at particularly high risk for sepsis due to multiple factors including immaturity, especially of the immune system, with decreased function of neutrophils and low concentration of immunoglobulins, prolonged hospitalization and frequent need of invasive procedures such as endotracheal intubation and intravascular catheterization (13). Neonatal sepsis occurs either due to vertical transmission by ascending contaminated amniotic fluid or horizontal transmission from direct contact with care providers or environmental source. Among surviving VLBW infants, 65% experience at least one suspected or culture-proven infection episode during their birth hospitalization and ~35% have culture-proven sepsis (14). Mortality following neonatal sepsis was reported to be up to 18% (13).

Studies investigating behavioral problems in former preterm infants differ greatly in design and assessment tool and thus many questions remain unanswered. The spectrum of behavioral problems in preterm infants has not been well-defined. While Johnson and Marlow described a “*preterm behavioral phenotype”* characterized by inattention, anxiety, and social difficulties (15, 16), the French population-based EPIPAGE study reported both externalizing (hyperactivity, peer problems) and internalizing problems (emotional symptoms) in very preterm infants at the age of 5 years (5). Behavioral problems have been described in preterm infants not only in infancy but well into adolescence (5, 16). Even preterm infants within the normal range of cognitive development are less likely to complete higher education than their term peers (17, 18) and more often need special educational support in mainstream schools. These problems persist with age (19) resulting in poorer adult occupational and social functioning (20).

In this study we aimed to investigate culture-proven neonatal sepsis as potential risk factor for behavioral problems in very low birth weight (VLBW) infants at preschool age. We hypothesized that neonatal sepsis may adversely influence behavioral outcome as this type of inflammatory processes have been described to be a crucial driving force for adverse outcomes in these patients (21).

## Methods

### Study population

This retrospective study included VLBW infants with a gestational age between 23^0/7^ and 31^6/7^ weeks and a birth weight <1,500 g, born in our level III neonatal center from 1st of January 2009 to 31st of May 2017. Infants who died during birth hospitalization or had major birth defects, i.e., identified genetic syndrome or congenital malformations were excluded from the study. Child Behavior Checklist (CBCL) questionnaires were distributed to the parents of the children included in the study when they were seen at the routine 5-year follow-up assessment in our outpatient clinic. Exclusion criteria for questionnaire distribution was the inability of the parents to comprehend the task instructions. Children completing the 5-year follow-up assessment who had an available CBCL parental report were eligible for data analysis. The study was conducted according to the guidelines of the Declaration of Helsinki and approved by the local ethics committee (EK-Nr. 1536/2020).

### Neonatal data

Neonatal data were collected from the medical records of the included infants: sex, gestational age, birth weight, neonatal sepsis (at least one episode of culture proven sepsis during birth hospitalization), severe intraventricular hemorrhage [IVH, grade 3 or higher, according to Deeg et al. (22)], severe retinopathy of prematurity (ROP, retinopathy of prematurity grade 3 or higher), bronchopulmonary dysplasia [BPD, oxygen dependency at 36 weeks post-menstrual age (23)], necrotizing enterocolitis [NEC Bell stage II or higher (24) with or without bacteremia] and socioeconomic status of the parents.

### Instruments and measures

Behavior at the age of 5 years was rated by the parents using the Child Behavior Checklist (CBCL) for ages 2–5 (25). The CBCL is a well-validated behavior rating questionnaire (25). It asks parents to rate 100 items on a 3-point Likert scale (0 = not true; 1 = somewhat/sometimes true; 2 = very/often true). The items describe specific kinds of behavioral, emotional, and social problems that characterize preschool children. Items are grouped into seven subscales—emotional reactivity, anxious/depressed, somatic complaints, withdrawn, sleep problems, attention, and aggression—as well as three summary scales of Total Problems, Internalizing and Externalizing Problems scores. In addition, five Diagnostic and Statistical Manual of Mental Disorders (DSM)-oriented syndrome scales can be extracted: Depressive problems, Anxiety problems, Autism spectrum problems, Attention deficit/hyperactivity (ADH) problems, and Oppositional defiant problems. From the DSM-oriented syndrome scale Autism spectrum problems and Oppositional defiant problems are reported in our study. Scores are reported as *t*-scores with mean of 50 and SD of 10. For the subscales, scores of 65–69 are considered borderline clinically significant and scores of 70 and above are considered clinically significant. For the Internalizing, Externalizing, and Total Problems scales, scores of 60 to 63 (~83rd−90th percentiles) are considered borderline clinically significant, and scores of ≥64 are clinically significant.

Socioeconomic status was calculated by means of a score reflecting both maternal education and paternal occupation (26). The score ranges from 2 to 12 points with a low score indicating high socioeconomic status and a high score indicating low status.

Cognitive performance in terms of intelligence (IQ) was assessed using the German version of the Kaufman Assessment Battery for Children (K-ABC) (27) administered by trained psychologists.

### Statistical analysis

Statistical analysis was performed using SPSS Statistics for Mac version 21.0. (Armonk, NY: IBM Corp). Qualitative data (describing the attributes or properties of a variable, categorizing it into classes) are shown as counts and percentages, while quantitative data are shown as mean and standard deviations (SD). First, we compared descriptive information and outcome information of infants with neonatal sepsis with infants without neonatal sepsis. Then, a multi linear regression model was used to isolate the effect of culture-proven neonatal sepsis on the behavioral outcome measured as CBCL scores of former preterm infants at 5 years of age. In the multivariate models, sex, gestational age, birth weight, severe IVH, severe ROP, NEC, BPD and socioeconomic status of the parents were considered as possible covariates. *P*-values <0.05 were considered statistically significant.

## Results

### Study population

**Figure 1:**
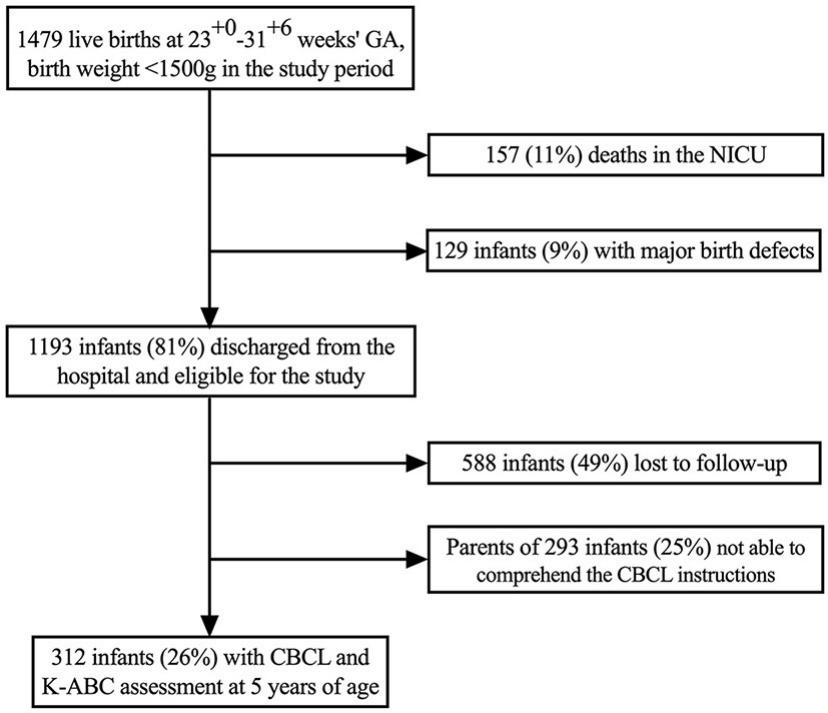
Study population.

In the study period 1,479 infants were born alive with a gestational age between 23^0/7^ and 31^6/7^ weeks and birth weight <1,500 g. Of these, 157 infants (11%) died in the neonatal intensive care unit and 129 infants (9%) had major birth defects and were therefore excluded from the study. Thus, 1,193 infants were eligible for CBCL and K-ABC assessment at 5 years of age. Of those, 588 infants (49%) were lost to follow-up because they did not participate in the 5 years follow-up at all and 293 infants (25%) could not be included because the parents were not able to comprehend the task instructions. Consequently, 312 infants (26%) had information on CBCL and K-ABC scores at 5 years of age and entered the final analysis (Figure 1).

Of the infants in the final analysis, 33 infants (11%) had experienced at least one episode of culture-proven sepsis during birth hospitalization. Detailed patient characteristics are given in Table 1. Infants with neonatal sepsis had a lower gestational age (*P* = 0.000), birth weight (*P* = 0.014) and a smaller head circumference (*P* = 0.035). They were more frequently mechanically ventilated (*P* = 0.024), had higher rates severe ROP (*P* = 0.006) and a longer duration of hospitalization (*P* = 0.001). Occurrence of NEC was slightly lower in the infants with neonatal sepsis (*P* = 0.045). At 5 years of age infants were similar for weight, length, neurocognitive outcome and socioeconomic status. The significantly lower head circumference in the infants with neonatal sepsis persisted at 5 years of age (*P* = 0.005).

**Table 1:** Patient characteristics.

	**Sepsis (*n*=33)**	**No Sepsis (*n*=279)**	***P*-values**
GA, weeks (mean ± SD)	25.64 ± 1.59	26.82 ± 2.05	0.000
BW, g (mean ± SD)	799.97 ± 229.30	913.19 ± 251.65	0.014
Gender, male (*n*, %)	20 (60.6)	136 (48.74)	0.269
HC, cm (mean ± SD)	24.17 ± 2.33	25.01 ± 2.15	0.035
APGAR 5 min (mean ± SD)	8.19 ± 0.91	8.38 ± 0.82	0.240
Severe IVH (*n*, %)	7 (21.21)	49 (17.56)	0.377
Severe ROP (*n*, %)	19 (57.57)	91 (32.61)	0.006
BPD (*n*, %)	10 (30.30)	49 (17.56)	0.152
NEC (*n*, %)	5 (15.15)	15 (5.37)	0.048
Mechanical ventilation, (*n*, %)	21 (63.63)	120 (43.01)	0.024
Birth hospitalization, days (mean ± SD)	104.12 ± 37.75	74.09 ± 30.59	0.001
Weight 5 years, kg (mean ± SD)	17.70 ± 3.11	18.75 ± 3.74	0.122
Length 5 years, cm (mean ± SD)	110.27 ± 5.46	111.92 ± 5.37	0.097
HC 5 years, cm (mean ± SD)	49.42 ± 2.09	50.45 ± 1.95	0.005
SES (mean ± SD)	5.13 ± 2.6	5.17 ± 2.35	0.937
K-ABC (mean ± SD)	91.97 ± 16.86	97.03 ± 16.23	0.100

*GA, gestational Age; BW, birth weight; HC, head circumference; IVH, intraventricular hemorrhage; ROP, retinopathy of prematurity; BPD, bronchopulmonary dysplasia; NEC, necrotizing enterocolitis; SES, socio economic status; K-ABC, Kaufmann assessment battery for children*.

### Neonatal sepsis and behavioral outcome at 5 years of age

The relationship between neonatal sepsis and behavioral outcome at the age of 5 years in VLBW infants was investigated by comparing the mean CBCL scores between the groups. Significant difference for raw uncorrected values could be observed for withdrawn behavior (*P* = 0.03), aggressive behavior (*P* = 0.04), internalizing behavior (*P* = 0.02) and CBCL total problems (*P* = 0.06) (Table 2).

**Table 2:** K-ABC and CBCL raw values comparison.

	**Sepsis**	**Mean**	**Std. deviation**	***P*-values**	**95% confidence interval**	
					**Lower margin**	**Upper Margin**
K-ABC	Yes	91.97	16.86	0.10	−11.23	1.10
	No	97.03	16.23			
Risk for autism	Yes	56.18	7.84	0.11	–.59	5.14
	No	53.91	6.20			
Oppositional behavior	Yes	53.12	5.29	0.19	–.41	3.43
	No	51.61	3.75			
Emotional reactivity	Yes	54.91	7.21	0.12	–.57	4.66
	No	52.86	5.11			
Anxiety/depression	Yes	54.88	6.47	0.11	–.50	4.22
	No	53.01	5.09			
Somatic complaints	Yes	54.39	6.14	0.62	−1.65	2.73
	No	53.85	6.05			
Withdrawn behavior	Yes	57.58	7.87	0.03	.22	4.91
	No	55.01	6.28			
Sleep disorders	Yes	53.36	6.74	0.36	−1.33	3.52
	No	52.27	3.81			
Attention/hyperactivity	Yes	53.88	6.29	0.24	–.96	3.62
	No	52.55	4.70			
Aggressive behavior	Yes	53.48	5.52	0.04	.078	4.08
	No	51.41	3.59			
Internalizing behavior	Yes	51.30	11.22	0.02	.50	8.16
	No	46.97	10.49			
Externalizing behavior	Yes	46.55	11.13	0.18	–.59	6.07
	No	43.80	8.95			
CBCL total problems score	Yes	36.48	7.81	0.06	–.08	4.12
	No	34.47	5.54			

When only VLBW infants with clinically relevant CBCL scores as defined in the Methods section were considered, an elevated risk for autism (*P* = 0.02), aggressive behavior (*P* = 0.03), and internalizing behavior (*P* = 0.01) persisted in the neonatal sepsis group (Table 3).

**Table 3:** CBCL clinical relevant scores.

	**Sepsis**	**Clinically relevant CBCL scores** **(*n*, %)**	***P*-values**
Risk for autism	Yes	6 (18.1)	0.02
	No	18 (6.4)	
Oppositional behavior	Yes	0 (0.0)	0.63
	No	4 (1.4)	
Emotional reactivity	Yes	1 (3.0)	0.95
	No	8 (2.8)	
Anxiety/depression	Yes	2 (6.0)	0.24
	No	7 (2.5)	
Somatic complaints	Yes	1 (3.0)	0.70
	No	17 (6.0)	
Withdrawn behavior	Yes	4 (12.1)	0.10
	No	14 (5.0)	
Sleep disorders	Yes	1 (3.0)	0.20
	No	1 (0.3)	
Attention/hyperactivity	Yes	1 (3.1)	0.71
	No	9 (3.2)	
Aggressive behavior	Yes	2 (6.0)	0.03
	No	1 (0.3)	
Internalizing behavior	Yes	6 (18)	0.01
	No	15 (5.3)	
Externalizing behavior	Yes	2 (6.0)	0.08
	No	3 (1.0)	
CBCL total problems	Yes	1 (3.0)	0.10
scores	No	1 (0.3)	

**Figure 2:**
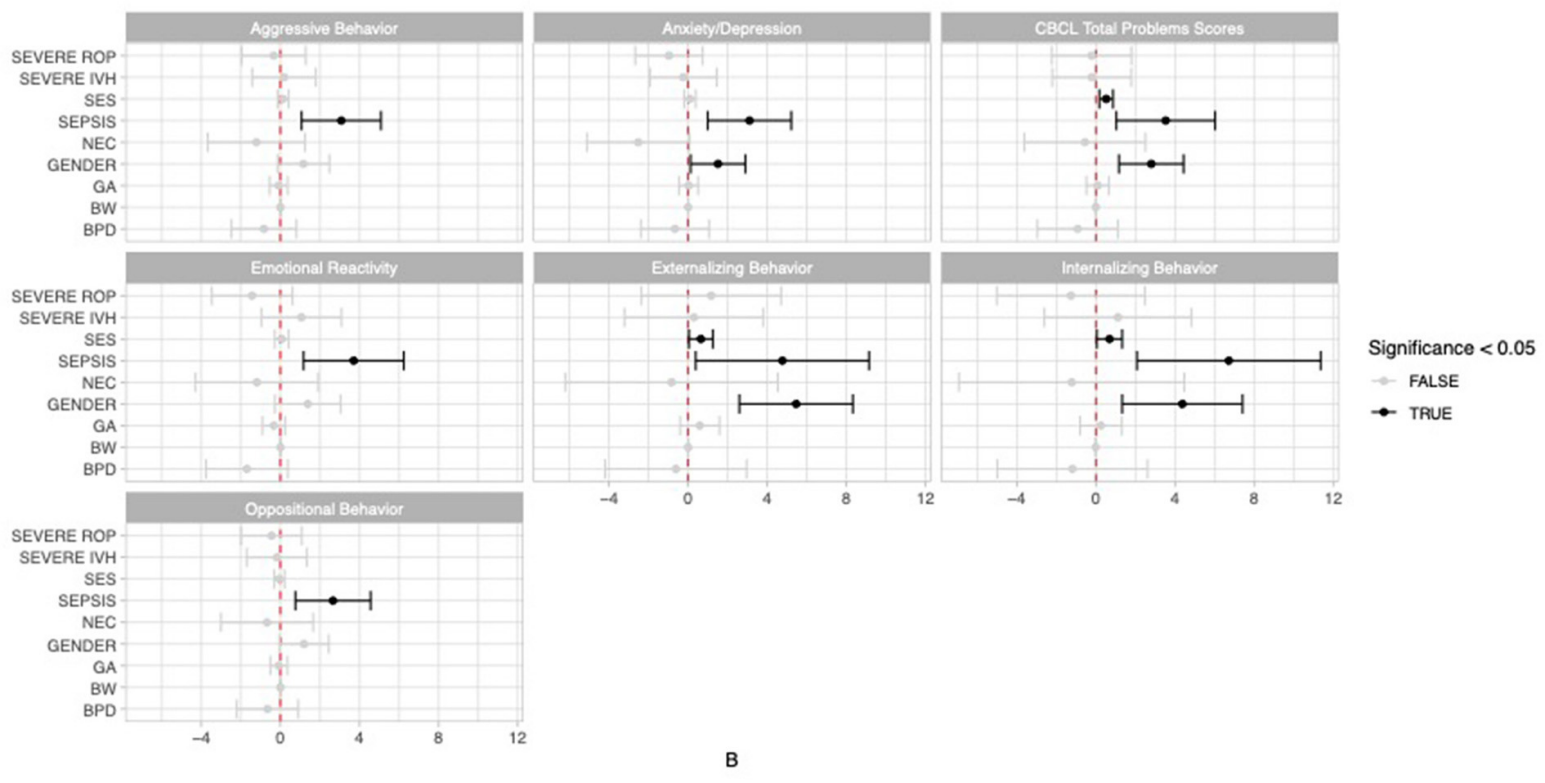
Graphical representation of the regression analysis investigating the association between neonatal sepsis and behavioral outcome in terms of CBCL scores of VLBWI at 5 years of age.

In the regression analysis, correcting for gender, gestational age, birth weight, severe IVH, severe ROP, BPD, NEC and socioeconomic status, neonatal sepsis was related with a significantly higher total CBCL problems score (*P* = 0.006) as well as with significantly more internalizing (*P* = 0.005) and externalizing behavioral problems (*P* = 0.033) in our cohort of VLBW infants with neonatal sepsis. Both low socioeconomic status and male gender were additional risk factors for a high total CBCL problem score (*P* = 0.003/*P* = 0.001), internalizing (*P* = 0.035/*P* = 0.005) and externalizing behavioral problems (*P* = 0.037/*P* = 0.000). The prevalent internalizing behavioral problems in VLBW infants with neonatal sepsis were emotional reactivity (*P* = 0.004) and anxiety/depression (*P* = 0.004), additionally influenced by male gender (*P* = 0.033). In contrast, the main externalizing behavioral problems were oppositional (*P* = 0.006) and aggressive behavior (*P* = 0.003) (Figure 2, Supplementary Table 1).

## Discussion

In this retrospective cohort study VLBW infants with culture-proven neonatal sepsis displayed a significantly increased rate of both externalizing and internalizing behavioral problems at preschool age compared to infants without diagnosis of neonatal sepsis during birth hospitalization. Low socioeconomic status and male gender were serious additional risk factors for both internalizing and externalizing problems in this cohort. Overall neurocognitive outcome was similar in VLBW infants with and without neonatal sepsis. The incidence of neonatal sepsis in our cohort was within the reported range for VLBW infants (21).

To our knowledge this is the first study analyzing the relationship between neonatal sepsis and the behavioral outcome in VLBW infants at preschool age. Neonatal sepsis is a major morbidity in VLBW infants because of its potentially severe course and potentially long-term sequalae. However, neonatal sepsis has been reported to be only a weak predictor of late death or neurosensory impairment in the smallest preterm infants (28), as opposed to BPD, brain injury and ROP (29). Still, it was reported that sustained elevations of acute inflammatory proteins during the first weeks of life were associated with a relevantly increased risk for various impairments of IQ and executive function in extremely preterm infants (30, 31). Animal models demonstrated that intrauterine and early post-natal inflammation pre-dispose the offspring to behavioral abnormalities and neuropsychiatric diseases such as depression, autism spectrum disorders and schizophrenia (32–34). These findings are of particular interest as behavioral problems in preterm infants do not seem to be related to typical neonatal cerebral abnormalities detectable with cranial ultrasound scan (5, 7, 35, 36). In fact, neuroimaging studies linked behavioral problems in preterm infants to more subtle cerebral lesion, in particular white matter lesions, which may be detected only with MRI (37, 38). Furthermore, increasing evidence suggests, that preterm infants may have altered microstructural connectivity (39, 40) as well as altered functional brain connectivity (41). Although not clarified yet, there may be a link between these alterations and the behavioral outcome of preterm infants as many studies report behavioral problems also in preterm infants without any neurocognitive impairment (5–7). Also, in our study neurodevelopmental outcome did not differ between the groups. White matter lesions have frequently been reported to occur in association with neonatal sepsis (37, 42–45). This fact may strengthen the findings of our study, where neonatal sepsis showed to be a significant risk factor for adverse behavioral outcomes in VLBW infants at preschool age. However, these neurophysiological and neuroanatomical associations have not been studied in detail so far and definitely warrant further investigation.

In our study neonatal sepsis was a risk factor for internalizing behavioral problems at the age of 5 years in former VLBW infants. These infants showed a significantly elevated risk for symptoms of depression, anxiety as well as for an elevated emotional reactivity. An internalizing behavioral phenotype has frequently been reported in preterm infants (5, 7, 46, 47) and may be of concern as it has been identified as a risk factor for psychiatric disorders already at school age (48) and also later in life (48, 49).

Besides elevated rates of internalizing behavioral problems, VLBW infants with neonatal sepsis also showed a higher rate of externalizing behavioral problems at preschool age, i.e., oppositional defiant and aggressive behavior. For conduct problems, so far, only a few studies have found an increased risk among very preterm or very low birth weight children (50, 51). Externalizing behavioral problems most often reported in preterm infants are hyperactivity/attention deficits (5, 15, 16), with inattention being more prevalent (16). In our study, similar to the study of Bartal et al. (7), we did not observe a higher risk for hyperactivity/inattention symptoms in VLBW infants with neonatal sepsis. In the clinical setting and particularly with young children, the diagnosis of inattention is often difficult compared to hyperactivity, which is more easily reported by parents. Also, inattention often becomes relevant only in the school setting and is therefore difficult to detect at preschool age.

Besides neonatal sepsis, also socioeconomic status was a relevant risk factor for both internalizing and externalizing problems in our cohort, however, to a lesser extent. This finding contradicts the frequently formulated statement that post-natal environment has a higher impact on behavioral outcome than neonatal morbidity (5–8).

Female gender showed to be protective against any type of behavioral problems at 5 years of age in our study population. The reason for this is not fully clarified yet but may be attributed in part to the fact that female preterms have a decreased risk for severe courses of infections as compared to males (52–54). Indeed, it has been shown before that sex influences the short-term behavioral outcome in very preterm infants in the context of inflammation (55). Brain development, including neural migration, synaptic pruning, developmental apoptosis, and glial maturation, differs between males and females and therefore, inflammatory insults occurring at the same gestational age may have different impacts (55, 56).

The present study has limitations worth mentioning. CBCL assessment was available only in German, which led to an elevated patient drop-out rate and may introduce a reporting bias. We compared two groups of VLBW infants. The comparison of our data to a reference sample of 5-year old healthy term children may have altered the results. There were significant differences between VLBW infants with and without neonatal sepsis, i.e., VLBW infants with neonatal sepsis had a lower gestational age and birth weight and more complications related to prematurity. However, our cohort showed similar growth and neurocognitive outcome at the age of 5 years.

In conclusion, our study supports previous findings of VLBW infants being vulnerable to behavioral disorders independent of their cognitive development. In contrast to former assumptions, the results of our study emphasize that not only post-natal environment but also neonatal morbidities are relevant for the behavioral outcome of VLBW infants at preschool age. Indeed, neonatal sepsis appeared to be a major risk factor for typical behavioral problems associated with prematurity, specifically internalizing and externalizing behavior. Therefore, prevention of neonatal sepsis remains an imperative goal during the primary hospitalization of very pre-mature infants. Furthermore, our results should encourage early screening for behavioral problems in the smallest preterm infants and initiation of early supporting measures in order to prevent later psychiatric disorders whenever possible and ensure academic success.

## Data availability statement

The raw data supporting the conclusions of this article will be made available by the authors, without undue reservation.

## Ethics statement

The study was conducted according to the guidelines of the Declaration of Helsinki and approved by the Ethics Committee of the Medical University of Vienna, Austria (EK-Nr. 1536/2020). Written informed consent from the participants' legal guardian/next of kin was not required to participate in this study in accordance with the national legislation and the institutional requirements.

## Author contributions

VG, SS, and CL were involved in collecting clinical data of the infants, while RF and AB collected outcome data of the included infants. KP and VG performed the statistical analysis and drafted the manuscript, helped by SS, AB, and CL. KP and VG conceptualized and supervised the study. All authors contributed to the article and approved the submitted version.

## Conflict of interest

The authors declare that the research was conducted in the absence of any commercial or financial relationships that could be construed as a potential conflict of interest.

## Publisher's note

All claims expressed in this article are solely those of the authors and do not necessarily represent those of their affiliated organizations, or those of the publisher, the editors and the reviewers. Any product that may be evaluated in this article, or claim that may be made by its manufacturer, is not guaranteed or endorsed by the publisher.
