# Supplementary material for: Neonatal sepsis is associated with behavioral abnormalities in very low birthweight infants at preschool age
Source: Front Pediatr. 2022 Jul 18;10:906379. doi: 10.3389/fped.2022.906379 (PMC9339780; doi:10.3389/fped.2022.906379)
Supplement: Supplementary file 1 [file Table_1.DOCX]

| **Suppl. Table 1:** Regression analysis* | | | | | | |
| --- | --- | --- | --- | --- | --- | --- |
|  | | Unstandardized Coefficients | | Sig. | 95,0% Confidence Interval for B | |
|  |  | B | Std. Error |  | Lower Bound | Upper Bound |
| **Oppositional Behavior** | Predictor |  |  |  |  |  |
|  | Gender | 1.197 | 0.631 | 0.059 | -0.049 | 2.443 |
|  | GA | -0.076 | 0.219 | 0.730 | -0.508 | 0.356 |
|  | BW | 0.001 | 0.002 | 0.637 | -0.003 | 0.004 |
|  | Severe_IVH | -0.180 | 0.771 | 0.816 | -1.703 | 1.343 |
|  | Severe_ROP | -0.452 | 0.777 | 0.561 | -1.987 | 1.082 |
|  | BPD | -0.652 | 0.787 | 0.409 | -2.206 | 0.902 |
|  | NEC | -0.675 | 1.181 | 0.569 | -3.008 | 1.658 |
|  | Sepsis | 2.661 | 0.964 | **0.006** | 0.758 | 4.563 |
|  | SES | -0.055 | 0.133 | 0.682 | -0.317 | 0.208 |
| **Emotional Reactivity** | Gender | 1.383 | 0.840 | 0.102 | -0.275 | 3.041 |
|  | GA | -0.333 | 0.291 | 0.255 | -0.908 | 0.242 |
|  | BW | -0.003 | 0.002 | 0.233 | -0.007 | 0.002 |
|  | Severe_IVH | 1.062 | 1.026 | 0.302 | -0.965 | 3.089 |
|  | Severe_ROP | -1.429 | 1.034 | 0.169 | -3.471 | 0.613 |
|  | BPD | -1.693 | 1.047 | 0.108 | -3.761 | 0.375 |
|  | NEC | -1.193 | 1.572 | 0.449 | -4.297 | 1.912 |
|  | Sepsis | 3.705 | 1.282 | **0.004** | 1.172 | 6.237 |
|  | SES | 0.065 | 0.177 | 0.712 | -0.284 | 0.415 |
| **Anxiety/Depression** | Gender | 1.509 | 0.701 | 0.033 | 0.125 | 2.893 |
|  | GA | 0.024 | 0.243 | 0.920 | -0.456 | 0.504 |
|  | BW | -0.002 | 0.002 | 0.250 | -0.006 | 0.002 |
|  | Severe_IVH | -0.242 | 0.857 | 0.778 | -1.933 | 1.450 |
|  | Severe_ROP | -0.971 | 0.863 | 0.262 | -2.676 | 0.733 |
|  | BPD | -0.668 | 0.874 | 0.446 | -2.394 | 1.058 |
|  | NEC | -2.525 | 1.312 | 0.056 | -5.117 | 0.066 |
|  | Sepsis | 3.104 | 1.070 | **0.004** | 0.990 | 5.218 |
|  | SES | 0.094 | 0.148 | 0.525 | -0.197 | 0.386 |
| **Aggressive Behavior** | Gender | 1.165 | 0.666 | 0.082 | -0.149 | 2.480 |
|  | GA | -0.087 | 0.231 | 0.707 | -0.543 | 0.369 |
|  | BW | 0.001 | 0.002 | 0.545 | -0.003 | 0.005 |
|  | Severe_IVH | 0.182 | 0.814 | 0.823 | -1.425 | 1.789 |
|  | Severe_ROP | -0.341 | 0.820 | 0.678 | -1.960 | 1.278 |
|  | BPD | -0.836 | 0.830 | 0.316 | -2.475 | 0.804 |
|  | NEC | -1.214 | 1.247 | 0.332 | -3.676 | 1.248 |
|  | Sepsis | 3.079 | 1.017 | **0.003** | 1.071 | 5.087 |
|  | SES | 0.135 | 0.140 | 0.337 | -0.142 | 0.412 |
| **Internalizing Behavior** | Gender | 4.373 | 1.540 | 0.005 | 1.331 | 7.414 |
|  | GA | 0.257 | 0.534 | 0.631 | -0.797 | 1.312 |
|  | BW | -0.006 | 0.004 | 0.155 | -0.015 | 0.002 |
|  | Severe_IVH | 1.110 | 1.883 | 0.556 | -2.607 | 4.828 |
|  | Severe_ROP | -1.261 | 1.897 | 0.507 | -5.007 | 2.485 |
|  | BPD | -1.185 | 1.921 | 0.538 | -4.978 | 2.609 |
|  | NEC | -1.227 | 2.884 | 0.671 | -6.922 | 4.468 |
|  | Sepsis | 6.723 | 2.353 | **0.005** | 2.077 | 11.368 |
|  | SES | 0.689 | 0.324 | 0.035 | 0.048 | 1.330 |
| **Externalizing Behavior** | Gender | 5.461 | 1.453 | 0.000 | 2.591 | 8.330 |
|  | GA | 0.591 | 0.504 | 0.242 | -0.404 | 1.586 |
|  | BW | -0.002 | 0.004 | 0.571 | -0.010 | 0.006 |
|  | Severe_IVH | 0.287 | 1.776 | 0.872 | -3.220 | 3.794 |
|  | Severe_ROP | 1.167 | 1.790 | 0.515 | -2.367 | 4.701 |
|  | BPD | -0.619 | 1.812 | 0.733 | -4.198 | 2.959 |
|  | NEC | -0.838 | 2.721 | 0.758 | -6.211 | 4.535 |
|  | Sepsis | 4.766 | 2.219 | **0.033** | 0.383 | 9.149 |
|  | SES | 0.644 | 0.306 | 0.037 | 0.039 | 1.248 |
| **CBCL Total Problems Scores** | Gender | 2.802 | 0.827 | 0.001 | 1.168 | 4.436 |
|  | GA | 0.093 | 0.287 | 0.746 | -0.474 | 0.660 |
|  | BW | -0.001 | 0.002 | 0.641 | -0.006 | 0.003 |
|  | Severe_IVH | -0.207 | 1.011 | 0.838 | -2.204 | 1.791 |
|  | Severe_ROP | -0.211 | 1.019 | 0.836 | -2.224 | 1.801 |
|  | BPD | -0.916 | 1.032 | 0.376 | -2.954 | 1.122 |
|  | NEC | -0.554 | 1.549 | 0.721 | -3.614 | 2.505 |
|  | Sepsis | 3.531 | 1.264 | **0.006** | 1.036 | 6.027 |
|  | SES | 0.522 | 0.174 | 0.003 | 0.178 | 0.866 |
| GA: Gestational Age; BW: Birth Weight; IVH: Intraventricular Hemorrhage; ROP: Retinopathy of Prematurity: NEC: Necrotizing Enterocolitis; SES: Socio Economic Status; CBCL: Child Behavior Checklist. *Regression presents results only for values that were found to be significant for Sepsis, the following CBCL subscales were therefore not included in the table: Risk for Autism, Somatic Complaints, Withdrawn Behavior, Sleep Disorders, and Attention/Hyperactivity. | | | | | | |
